# Eosinophilic Granulomatosis With Polyangiitis: Dissecting the Pathophysiology

**DOI:** 10.3389/fmed.2021.627776

**Published:** 2021-02-24

**Authors:** Filippo Fagni, Federica Bello, Giacomo Emmi

**Affiliations:** Department of Experimental and Clinical Medicine, University of Firenze, Firenze, Italy

**Keywords:** Eosinophilic Granulomatosis with Polyangiitis, Churg-Strauss syndrome, eosinophils, hypereosinophilic syndromes, ANCA-associated vasculitis, neutrophils, myeloperoxidase, EGPA classification

## Abstract

Eosinophilic Granulomatosis with Polyangiitis (EGPA) is a rare multisystemic disease classified both amongst hypereosinophilic disorders and ANCA-associated vasculitis. Vessel inflammation and eosinophilic proliferation are the hallmarks of the disease and main effectors of organ damage. Two distinct disease phenotypes have classically been described according to ANCA-status: the ANCA-negative subset with eosinophil-driven manifestation and the ANCA-positive one with vasculitic manifestations. An analogous dichotomization has also been backed by histological findings and a distinct genetic background. EGPA is typically consider a Th2-mediated disease and blood and tissue eosinophilia represent the cornerstone of diagnosis. Besides, ANCA are known for inducing endothelial injury and vascular inflammation by activating the circulating neutrophils. Thus, the pathogenesis of EGPA seems to be mediated by two coexisting mechanisms. However, the verbatim application of this strict dualism cannot always be translated into routine clinical practice. In the present review we describe the current knowledge on the eosinophilic and ANCA-mediated aspects of EGPA pathogenesis. Finally, we review the rationale of the currently proposed EGPA dichotomy and future research perspectives.

## Introduction

Eosinophilic Granulomatosis with Polyangiitis (EGPA) is a rare disease characterized by granulomatous and eosinophil rich inflammation and systemic necrotizing vasculitis affecting small-to-medium sized vessels. EGPA occurs in patients with asthma and peripheral and tissue eosinophilia, and ~30% of the patients present antineutrophil cytoplasm antibodies (ANCA) mainly specific for myeloperoxidase (MPO) (1). The disease is unique in its genre as it combines asthmatic manifestations with hypereosinophilic disorders and ANCA-associated vasculitis (AAV) features. Therefore, a full comprehension of its pathophysiology still lies beyond our reach.

An increasing amount of evidence indicates that EGPA's clinical phenotypes tends to segregate according to ANCA-status, as the major eosinophil-driven complications are most frequently found in the ANCA-negative subset of EGPA, namely lung infiltrates, myocardiopathy, and gastrointestinal manifestations. In contrast, MPO-ANCA-positive patients present a more “vasculitic phenotype,” which comprises palpable purpura, peripheral neuropathy, rapidly progressive glomerulonephritis and, rarely alveolar hemorrhage (2, 3) (Table 1). An analogous dichotomy is also supported by histological findings, as biopsy-proven vasculitis is found more frequently in ANCA-positive patients than in ANCA-negative ones, whereas eosinophilic infiltrates and granulomas are found with a similar frequency in the two groups (24).

**Table 1:** Prevalence of EGPA clinical characteristics according to ANCA-status and biomarkers of vasculitic and eosinophilic activity^*^.

**Main EGPA clinical manifestations**	**ANCA+**	**ANCA-**
Asthma	95%	93.1%
ENT involvement	68.8%	54.5%
Lung involvement *(all kinds)*^†^	60.6%	84.2%
Alveolar hemorrhage	16%	3.2%
Skin involvement *(all kinds)*^#^	50%	40.9%
Palpable purpura	30%	17.6%
Peripheral neuropathy	69.3%	50.6%
CNS involvement	9.7%	16.6%
Renal involvement *(all kinds)*^§^	33.3%	13.1%
NCGN	23%	2.3%
Heart Involvement	14.5%	32.6%
Gastrointestinal involvement	26.2%	23.6%
**Potential biomarkers of disease activity**		
**Biomarkers of vasculitic activity**	**Biomarkers of eosinophilic activity**	
**ANCA**	**Eosinophils**	
Patients with persistently elevated ANCA titer, or with re-appearance of ANCA or increase in serum ANCA levels present an higher risk of vasculitis relapse (4).	Absolute eosinophil count correlates with disease activity and risk of relapse with moderate sensitivity and specificity. In untreated EGPA. However, treatment with glucocorticoids and immunosuppressants may be a source of confoundment (5).	
**Urinary-MCP-1**	**IgG4**	
MCP-1 is a chemokine that attracts circulating monocytes in renal glomeruli. Urinary MCP-1 levels are elevated in patients with active renal vasculitis and correlate renal disease activity and response to therapy (6).	Th2 cytokines promote Ig class switching to IgG4. Preliminary studies suggested that IgG4 levels may reflect EGPA disease activity. However, there is conflicting evidence from longitudinal studies on serum IgG4 and IgG4/IgG ratio. Routine determination is not yet recommended (7, 8).	
**Urinary soluble-CD163**	**CCL26/Eotaxin-3**	
Urinary sCD163 is released by crescent macrophages. Its detection correlates with necrotizing crescentic glomerulonephritis and it may represent a biomarker for active renal vasculitis (9). The combination of elevated usCD163 plus either elevated uMCP-1 or new/worse proteinuria improved the positive likelihood ratio of active renal vasculitis (10).	Eotaxin-3 is a highly eosinophil-specific chemoattractant. Preliminary studies suggested that serum eotaxin-3 levels may be a sensitive and specific marker for active EGPA. However, there is conflicting evidence from longitudinal studies. Routine determination is not yet recommended (7, 11).	
**Serum soluble CD25 and urinary soluble CD25**	**CCL17/TARC**	
CD25 is a T cell activation marker. Measurement of ssCD25 and usCD25 supports usCD163 in the detection of active renal vasculitis (12).	CCL17/TARC is a chemokine that can induce the chemoattraction of activated Th2 cells. Preliminary studies suggested that CCL17/TARC levels may reflect EGPA disease activity. However, there is conflicting evidence from longitudinal studies. Routine determination is not yet recommended (7, 13).	
**Alternative complement pathway**	**ECP**	
Serum C3a, C5a, soluble C5b-9, and Bb fraction correlate with disease activity in MPO-ANCA positive renal vasculitis (14). Avacopan (C5aR antagonist) is being explored as a potential therapeutic target, but specific data on EGPA are lacking (15).	ECP is a cardiotoxic and neurotoxic eosinophil granule protein. It was correlated with EGPA disease activity and eosinophil count in preliminary studies. A significant independent correlation with atherothrombotic risk in EGPA was also described (16, 17).	
**Markers of B cells activation**	**Periostin**	
Markers of B cell activation (BAFF) and B cell repopulation after rituximab therapy (high frequencies of switched memory B cells and circulating plasmablasts—CD27^+^CD38^hi^) have been shown to correlate with AAV disease activity and relapse. However, specific data on EGPA are missing. Further studies are required to determine whether they may become potential biomarkers for EGPA vasculitic activity (18–22).	Periostin has been implicated in eosinophil function and recruitment. Serum periostin was modestly associated with EGPA disease activity and was higher in EGPA compared to healthy controls and asthmatics in a preliminary study (23).	

* *Percentages for every clinical feature were obtained by combination (weighted average) of data from Sinico et al. (2), Sablé-Fortassou et al. (3), and Comarmond et al. (24); data on NCGN were obtained by combination (weighted average) of data from Sinico et al. (2) and Sablé-Fortassou et al. (3)*.

† *Lung involvement (all kinds) comprises migratory lung infiltrates, lung nodules, chest pain, pleural effusion, and alveolar hemorrhage*.

# *Skin involvement (all kinds) comprises urticaria, purpura, livedo, subcutaneous nodules, and ulcers*.

§ *Renal involvement (all kinds) comprises raise in creatinine serum levels, proteinuria > 0,4 mg/24 h, haematuria> 10 red blood cells/high power field, and NCGN*.

*EGPA, eosinophilic granulomatosis with polyangiitis; ANCA, antineutrophil cytoplasmic antibodies; ENT, ear-nose-throat; CNS, central nervous system; NCGN, necrotizing crescentic glomerulonephritis; u-MCP-1, urinary-monocyte chemoattractant protein 1; ECP, eosinophil cationic protein; BAFF, B cell-activating factor*.

The dualism between ANCA-positive and ANCA-negative EGPA is also supported by genetic background. A recent genome wide association study (GWAS) found differential association of genetic variants between the two serological subsets. MPO/ANCA-positive EGPA has a significant association with HLA class II DQ haplotype, which is shared with the other MPO-AAV (i.e., microscopic polyangiitis, MPA), while ANCA-negativity is associated with GP33 and IL5/IRF1 loci, indicating a possible mucosal/barrier dysfunction origin (25).

The pathogenesis of the disease also results from the complex interaction among innate and adaptive immunity, including eosinophils, neutrophils, T-helper lymphocytes, and B lymphocytes (26).

Based on these premises, the present review will focus on untangling the interactions between the two main pathogenic processes in EGPA (i.e., eosinophilic vs. vasculitic). The validity of the current two-faced model of the disease will also be examined.

## EGPA as an Eosinophilic Disorder

Eosinophils are granulocyte innate immune cells that have classically been described in allergy, host defense against parasites, myelo- and lympho-proliferative disorders, and in autoimmune diseases. Particularly, blood and tissue eosinophilia represent the diagnostic cornerstone of EGPA, making it the prototype of eosinophilic vasculitis (5). From a pathophysiological point of view, EGPA shares intrinsic mechanisms with allergy and anti-helminthic response (27–30). It is characterized by the *en masse* polarization of T helper lymphocytes toward a Th_2_ phenotype, the upregulation of eosinophil-selective eotaxin chemokines (particularly eotaxin-3), and an increased secretion of eosinophilotropic cytokines [i.e., interleukin (IL)-4, IL-5, IL-9, IL-13, and IL-25] (28, 31). So-called “allergic granulomas,” consisting of palisading giant cells surrounding a core of necrotizing eosinophils, are also a distinctive histopathological feature of EGPA and are a sign of chronic eosinophilic inflammation which have also been described within persistent helminthic infections (29). Eosinophil-mediated organ damage is a shared feature of both EGPA and hypereosinophilic syndrome (HES), and clinical aspects overlap considerably (32). From a pathogenic standpoint however, a myeloid or lymphoid clonal origin can be detected in nearly half of HES (32, 33), and sensitivity to imatinib has been reported in a number of FIP1L1-PDGFRA (F/P)-negative HES patients bearing other novel fusion genes (34–36). Nonetheless, imatinib also anecdotally showed efficacy in F/P-unmutated EGPA, while a F/P-positive EGPA patient was reported, suggesting possible shared pathogenic mechanisms with HES (37, 38).

### Eosinophils' Cytotoxicity

**Figure 1:**
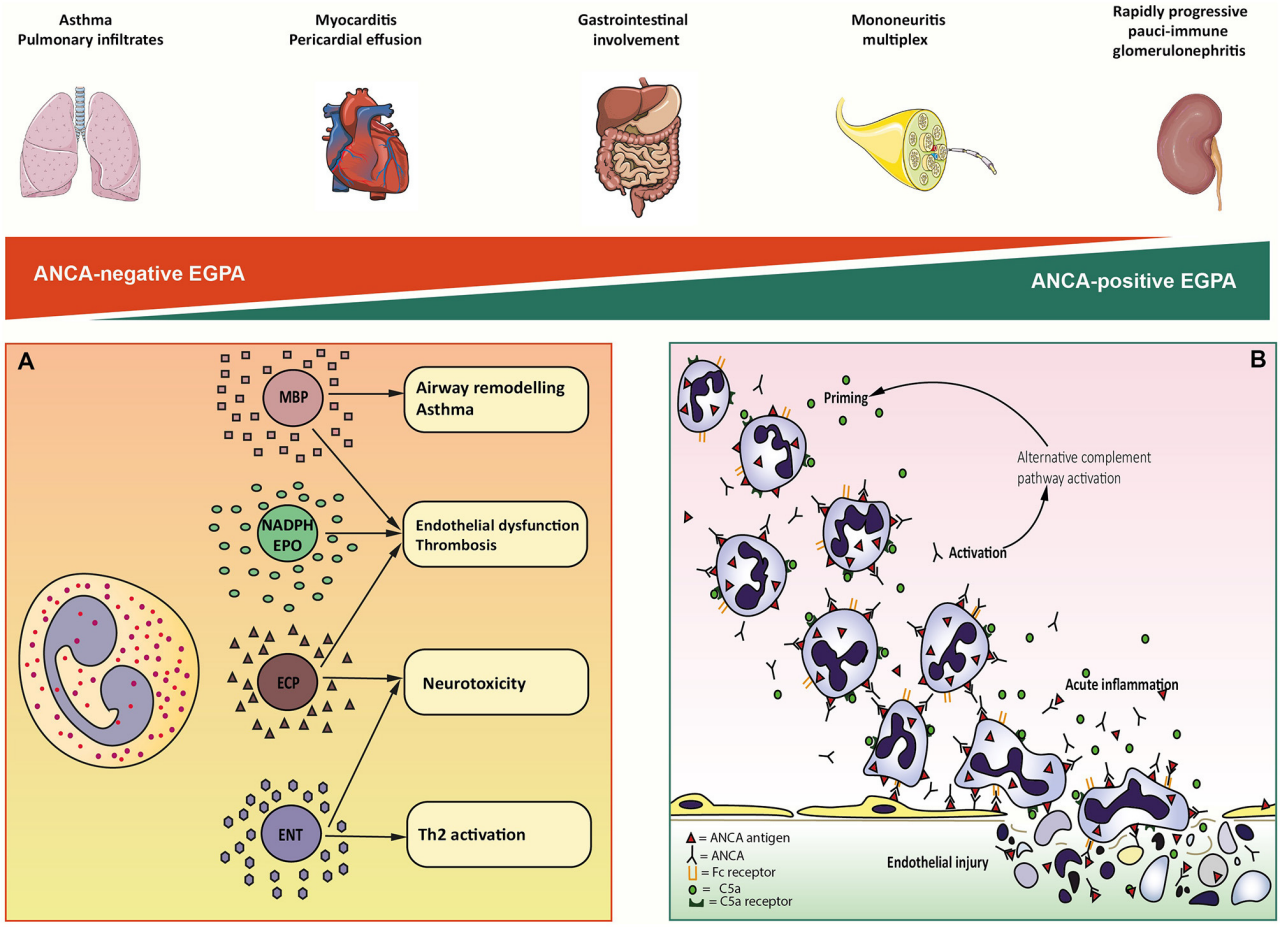
Overview of eosinophil-mediated and ANCA-mediated mechanisms of EGPA immunopathogenesis. On top, a schematic representation of the spectral distribution of clinical features in MPO-ANCA-negative (red triangle) and MPO-ANCA-positive EGPA (green triangle). At the extreme ends of the spectrum, lung involvement and rapidly progressive pauci-immune glomerulonephritis come as the result of predominantly eosinophilic and vasculitic mechanisms of organ damage, respectively. In contrast, the other clinical manifestations of EGPA result from the combination of the two processes. **(A)** Eosinophil-mediated pathogenesis of EGPA. Eosinophil granule proteins are highly cationic compounds that act synergically to mediate the cell's cytotoxicity. Mayor Basic Protein (MBP) is associated with airway remodeling and asthma, fibrogenesis, and procoagulant activity. NADPH-oxidase (NADPH) and Eosinophil Peroxidase (EPO) generate high quantities of reactive oxygen species that contribute to endothelial dysfunction and thrombosis. Eosinophil Cationic Proteins (ECP) is implied in cardiac toxicity, procoagulant activity, and nerve fibers degeneration. Eosinophil Neurotoxin (ENT) has marked neurotoxic potential *in vivo*. **(B)** Putative sequence of neutrophil-mediated endothelial injury. Circulating neutrophils get primed for ANCA activation by inflammatory cytokines and C5a complement factor. Priming induces the exposition on neutrophils cell-surface of ANCA antigens. Circulating ANCA bind to ANCA-antigens through F(ab)2 fragment and activate neutrophils by interaction with Fc receptor. Activated neutrophils release cytotoxic enzymes and factors that activate the alternative complement pathway, producing C5a that further enhances neutrophils priming. ANCA-activated neutrophils marginate and penetrate the vessel wall, where they undergo respiratory burst, degranulation, NETosis, and necrosis causing endothelial damage.

Eosinophils exhibit a wide spectrum of cytotoxicity, that is mediated by an array of enzymes stored in cytoplasmic granules, each of which associated with distinct type of clinically observable organ damage (39) (Figure 1A). Cardiac involvement is the major cause of mortality and morbidity in EGPA and has been widely associated to eosinophilia (40–42). *In vitro* evidence suggests that cardiotoxicity is mainly mediated by eosinophilic cationic protein (ECP) by altering the membrane sodium permeability of cardiomyocytes and inhibiting mitochondrial respiration (41). ECP also mediates fibrogenesis by inducing the release of fibrogenic cytokines transforming growth factor β (TGF- β), IL-1α, and IL-1β (43). Consistently, the presence of eosinophilic infiltrates and granule proteins has been widely documented in fibrotic tissues, including endomyocardial biopsy specimens of patients with EGPA (44, 45). The neurotoxic properties of eosinophils are clinically evident in the form of axonal neuropathy, a frequent finding in EGPA. Histologically, this relates to the presence of infiltrating eosinophils in the endoneurium and epineural vessels of EGPA patients (46). Fiber damage is probably due to the activity of eosinophilic neurotoxin (ENT) and ECP, which have been found to induce it *in vivo* (47). Interestingly, ENT acts as activating factor for myeloid dendritic cells by triggering the Toll-like receptor 2 (TLR2)–MyD88 signaling pathway, which is associated with the secretion of Th_2_ type interleukins (48). Thus, ENT might have similar properties to an endogenous alarmin, alerting the immune system for preferential enhancement of antigen-specific Th_2_ response (48). Airway remodeling, subepithelial fibrosis, and ciliated cells destruction have been linked to the activity of major basic protein (MBP) *in vivo* (49). MBP is an abundant granule protein that can induce histamine release from basophiles, superoxide generation by alveolar macrophages, and fibrogenesis through TGF-β signaling (39, 50). Consistently, toxic-range concentrations of MBP were found in sputum and pleural fluid from asthmatics (51). An emerging aspect in eosinophils pathophysiology is their ability to induce a prothrombotic microenvironment on the endothelium. This clinically relates to an increased risk of arterial and venous thrombosis, which can be observed in EGPA (52, 53). Eosinophils can autonomously generate thrombin and induce tissue factor exposure on endothelial cells. This leads to increase platelet adhesion to the vascular wall and thrombus growth (54). Intravascular eosinophil activation also induces the formation of eosinophil extracellular traps, which can be found in human thrombi and could have a potential role in injury-related thrombosis (55). Prothrombotic alterations are also linked to ECP- and MBP-mediated interference with the coagulation cascade and to aberrant eosinophil-derived reactive oxygen species (ROS) production (56–58). Eosinophil NADPH-oxidase works in concert with eosinophil peroxidase (EPO) to generate high levels of ROS from H_2_O_2_, which in turn interact with endotheliocytes' cellular signaling to upregulate genes for adhesion molecules, tissue factor, and vasoactive substances (58, 59). Most importantly, eosinophil-derived ROS promotes lipoperoxidation, thereby contributing to atheromatous plaque formation and destabilization (55).

### Genetic Background of Eosinophilia

Our knowledge of eosinophils' biology allowed their identification as the sole perpetrators of non-vasculitic clinical manifestations in EGPA. However, our current understanding of the primitive pathological alterations underlying the triggers and drivers of eosinophilic inflammation in EGPA is still incomplete (25).

A predisposition based on immunogenetic factors is known. EGPA is associated to HLA alleles DRB1^*^04 and ^*^07 and with HLA-DRB4, suggesting a strong link with CD4+ T-lymphocyte activation (60, 61). Furthermore, functionally relevant variations of the IL-10 gene promoter were associated with EGPA in general (62), whereas IRF1/IL5 and GPA33 genes variants were associated with MPO-ANCA-negative EGPA (25). Interestingly, IL-10 and IRF1/IL5 both relate to eosinophilic inflammation. IL-10 is pivotal for the activation of the Th_2_pathway, while IRF1/IL5 can interact with the regulatory regions of IL-4 and IL-5 (25, 62). Analyzed IRF1/IL5 variants were associated with an increased risk to develop EGPA, higher eosinophils, and severe asthma (25). Intriguingly, GPA33 encodes a surface glycoprotein that contributes to intestinal and bronchial mucosal function, hinting at a role of barrier dysfunction and innate immunity in disease development (63).

### Adaptive and Innate Immune Response and Eosinophils

Ultimately, a dysfunctional communication between innate and adaptive type 2 immunity seems to be at the root of eosinophilia in EGPA. EGPA is generally considered as a Th_2_-response-mediated disease due to the high eosinophil activity and the characteristically elevated serum levels of Th_2_cytokines (7, 28). Amongst them, IL-5 has the most relevant impact on the differentiation, proliferation, and survival of eosinophils and proved to be a promising therapeutic target (64). Indeed, the surface expression of the IL-5 receptor (CD125/CD131) is a key terminal step in eosinophil haematopoiesis and circulating IL-5 levels regulate the mobilization of eosinophils from the bone marrow (65). At a tissue level, the Th_2_ differentiation marker CD294 is abundantly present in biopsies from EGPA patients (13), and a Th_2_-dominant transcriptomic profile (STAT3, STAT6, GATA3, IL10, and IL4) was also described in bronchoalveolar lavage (66). Tissue and circulating eosinophils in EGPA also secrete IL-25, a potent eosinophilotropic cytokine that induces their own proliferation and Th_2_-response, thereby maintaining a vicious cycle (67). Eosinophils is EGPA are also impaired qualitatively. This is revealed by an increase in surface-expressed eosinophil activation CD69 and CD25, and by evidence of dysfunctional apoptotic pathways (68, 69). EGPA is associated with variants of the apoptosis-controlling BCL2L11 and MORRBID genes (25), the latter of which is dysregulated in hypereosinophilic syndromes (70). Furthermore, several proapoptotic genes (BCL2L13, CASP2, and CARD4) were found underexpressed in the eosinophils of EGPA patients (69), and high circulating levels of soluble CD95 (an inhibitor of Fas-mediated apoptosis) were also described (71). Finally, although their role has yet to be fully characterized, it must be mentioned that IL-5-producing innate lymphoid cells type 2 (ILC2) were also found elevated in EGPA and their blood concentration correlates with eosinophil count and disease activity (72). ILC2s are strategically embedded in peripheral tissues and orchestrate the crosstalk between epithelial cells and the immune system. Their activity has been linked to the initiation of type-2 immune responses *via* IL-5 and IL-13 production, eosinophils and mast cells recruitment, and M2 macrophage polarization (73). Although their real contribution to EGPA still needs to be investigated, their privileged position as an interface between innate immunity and the adaptive Th_2_ response could be promising for future research developments (74, 75).

## EGPA as a Vasculitic Disorder

### Animal and Human Models of ANCA-Mediated Vascular Inflammation

ANCA prevalence in EGPA varies from 30 to 40%. Of the EGPA patients that test positive for ANCA, 90-to 100% have MPO-ANCA specificity. Although the pathogenic role of anti-MPO ANCA has not been overtly demonstrated in EGPA, it is presumed that similar mechanisms to the ones known in MPA occur. Several animal models have demonstrated the direct noxious role of ANCA toward endothelial cells and their key interaction with neutrophils in vasculitis pathogenesis as the cause of necrotizing-crescentic glomerulonephritis (NCGN) and pulmonary hemorrhage (76). The pathogenic potential of MPO-ANCA has been documented either by injection of anti-MPO IgG in mice (77), by injection of splenocytes containing anti-MPO positive B-cells in Rag2^−/−^ mice (which lack B and T cell responses) (77) or by transplanting bone marrow that contain MPO-positive myeloid cells in irradiated MPO^−/−^ mice previously immunized with MPO antigen (78).

However, the relationship between ANCA and EGPA manifestations appears more complex as autoantibody titer does not always correlate with disease severity and ANCA can persist in remission phases or re-appear without clear disease activity (79, 80). Furthermore, a percentage of patients with vasculitic manifestations test negative for ANCA (81, 82), and conversely low-titer non-pathogenic ANCA have been described in healthy individuals (83, 84). These elements suggest that, despite ANCA being directly pathogenic, not all ANCA appear effectively involved. Indeed, more than 20 MPO epitopes have been identified. Antibodies to MPO specific for disease active phases were proven to be strong ROS inducers from neutrophils, whereas antibodies in healthy individuals evoked a poor neutrophilic response (85).

Recently anti-lactoferrin antibodies have been described in EGPA patients, but not in GPA and MPA, and have been directly correlated to disease activity (86). However, anti-lactoferrin antibodies can also be found in other several autoimmune diseases. Thus, whether these autoantibodies are effectively pathogenic or represent just an epiphenomenon is still unknown. Nevertheless, their detection may suggest the presence of ANCA directed toward alternative epitopes, which we are not able to identify yet.

Studies on mouse models also showed that disease manifestations could be limited by modification of ANCA IgG glycosylation on the Fc fragment (87), affecting FcγR-antibody interaction or through inhibition of p38 mitogen-activated protein kinase (MAPK), which is thought to prime and activate neutrophils (88). The *scenario* proposed for ANCA-mediated vascular injury starts with neutrophil priming by circulating inflammatory cytokines (Figure 1B). Once primed, neutrophils expose a small amount of ANCA antigens (normally sequestered in the cytoplasmic granules) on cell surface. ANCA F(ab)2 fragment binds to surface antigens, while the Fc fragment interacts with FcγRIIa and FcγRIIIb, triggering the respiratory burst (89). Activated neutrophils penetrate the vessel wall and release ROS and toxic enzymes causing the necrosis of endothelial cells and adjacent matrix. In addition, monocytes can be similarly activated by ANCA. Activated monocytes contribute to further neutrophil recruitment and activation and to granulomatous lesions formation. Several studies have also suggested the participation of alternative complement pathway in ANCA-induced inflammation (90, 91). Activated neutrophils release factors which activate the alternative complement pathway, resulting in the generation of C5a fragment, which in turn attracts neutrophils at the site of inflammation and primes the incoming neutrophils for ANCA activation (92).

### ANCA and Adaptive Immune Responses

Adaptive immune responses also appear to be involved. A significant contribution comes from T cells, which can be found in NCGN biopsy specimens and in EGPA granulomatous lesions. It has been shown that activated neutrophils do not only cause endothelial injury but also deposit MPO-antigen in glomeruli and in the basal membrane. Anti-MPO CD4+ T cells recognize the planted glomerular MPO and amplify the immune-mediated damage (93, 94).

Increased frequencies of Th17 lymphocytes in peripheral blood samples from EGPA patients are reported during relapse of vasculitic manifestations (95). It has been proposed that MPO-ANCA activated neutrophils, through IL-6, IL-17, and IL-23 production create a permissive environment for T cells to differentiate toward a Th17 phenotype (96, 97). This generates an amplification loop in which Th17 lymphocytes promote neutrophils recruitment and activation (98).

### Origin of Pathogenic-ANCA

If it is almost ascertained that ANCA play a direct role in the pathogenesis of vasculitic manifestations, the initial events leading to tolerance breakdown and to autoantibodies production remain still enigmatic. Since GWAS studies have linked MPO production with specific HLA haplotypes, it is possible to theorize that in patients with a genetically determined predisposition in antigen recognition, ANCA are produced as an initially appropriate immune response, that would lately transform into an aberrant autoimmune process. The proposed antigens include microbial peptides (99, 100), drugs (namely hydralazine, minocycline, propylthiouracil, and levamisole-adulterated cocaine) (101) or endogenously produced antisense transcripts of MPO or PR3 genes (102, 103). However, such a process has never been proved neither for MPO or PR3 ANCA.

Another factor that may influence ANCA production is epigenetic modification of MPO expression, which appear disrupted in AAV patients, potentially contributing to overexpression of ANCA antigens on neutrophil surface (104). Central loss of tolerance toward MPO was proposed to be involved in ANCA production. MPO is expressed in an AIRE-dependent manner in the thymus and its expression is involved in the central deletion of potentially autoreactive anti-MPO CD4+ T cells (105). Moreover, a defective activity both in regulatory T cells (Tregs) and regulatory B cells (Breg) have been reported in EGPA (106–108). From a functional point of view, it appears that the depletion of Tregs and Bregs, which normally would suppress immune responses, could facilitate the production of ANCA from effector B cells. The role of Bregs and autoreactive B cells is also suggested by the efficacy of rituximab (anti-CD20 antibody) (109, 110). In patients treated with rituximab, the peripheral Breg restoration rate correlates with a more effective remission of the vasculitic process (111). Finally, recent studies have focused on the possible role of a mechanism known as NETosis in ANCA production. Neutrophil extracellular traps (NETs) are a framework of chromatin fibers and antimicrobial proteins, including MPO that are released from dying neutrophils as a defense mechanism against microbes. Even in the absence of infective stimuli, the formation of NETs is enhanced in AAV patients compared to healthy controls. NETs are thought to facilitates ANCA developing by presenting antigens to the adaptive immune system (112, 113).

Intriguingly, ANCA have been detected in sputum samples of EGPA patients, irrespective to their serum ANCA-status. Despite unknown specific targets, sputum autoantibodies induced both neutrophil and eosinophil extracellular traps *in vitro*, suggesting their possible pathogenicity (114). It is tempting to speculate that sputum-ANCA may preceded the development of serum-ANCA positivity in a subset of EGPA patients.

Despite the presented evidence, the role of ANCA in EGPA pathogenesis is still under query. ANCA-positive EGPA patients suffer more, albeit not excursively, from vasculitis symptoms, and contrarily, not all patients which developed vasculitic manifestations display ANCA positivity.

Furthermore, the clinical differences of EGPA with other MPO-positive vasculitis contribute to the puzzling picture. Indeed, in ANCA-positive EGPA the prevalence of renal involvement varies from 27 to 51% (2, 3, 24), while in MPA, NCGN involves almost all patients (70 to 90–100%) (115, 116). A similar gap may be found also for alveolar hemorrhage, whose prevalence varies from 2 to 16% in ANCA-positive EGPA patients (24, 117, 118) against 12–30% in MPA patients (116, 119). The reasons for this attenuated vasculitic phenotype in MPO-positive EGPA compared with MPA remains undercover.

## What is Missing in EGPA Dichotomy?

Progressive improvements in our clinical and pathophysiological understanding of EGPA have reflected into significant advances in the early diagnosis and treatment of the disease. However, the processes through which molecular- and cellular-scale disease mechanisms translate into macroscopic clinical changes still needs to be thoroughly elucidated.

While the pathophysiology of EGPA could be dichotomised as either “eosinophil-driven” or “ANCA-driven,” the same principle cannot be applied clinically. EGPA has been classically described to evolve through a prodromic allergic phase characterized by asthma and rhinosinusitis, an eosinophilic phase with blood and tissue eosinophilia, and a vasculitic phase with organ involvement secondary to small-vessel vasculitis. However, these phases partially overlap and may not appear in such a defined order.Besides, clinical features typical of vasculitis, such as glomerulonephritis or neuropathy, can be observed in both ANCA-negative and ANCA-positive EGPA regardless of the disease phase (81). Moreover, some clinical manifestations including cardiomyopathy and neuropathy could come as a result of the overlapping influence of eosinophilic infiltration and vasculitis (120, 121). These phases and the varied pathological findings suggest that the pathophysiology of the disorder might evolve over time. We could therefore speculate that EGPA may represent a spectrum of disease phenotypes that cannot be perfectly encapsulated by a single serological or clinical descriptor (Figure 1). For instance, a clear phenotypic subdivision is not commonly observed in the clinical practice. Cluster analysis performed on the basis of clinical features supports this observation, as it failed in demonstrating an explicit *dichotomy* and an intermediate phenotype with frequent renal, gastrointestinal, and cardiovascular involvement emerged (122).

Furthermore, the two subgroups share clinical, pathological and genetic features. Indeed, patients display severe asthmatic manifestations, and a percentage has a history of allergy, independently from ANCA-status. Eosinophilia and granulomatous lesions are found with analogous frequencies in both subgroups and several genetic variants associated with asthma and elevated eosinophil levels are shared (25). We can therefore speculate that EGPA is primarily caused by intrinsic eosinophil dysfunction, upon which a group of genetically predisposed patients, which present HLA-DQ variant, develop anti-MPO autoantibodies in response to an unidentified stimulus.

Additionally, the clinical value of ANCA-positivity should not be overestimated. Since MPO targeted epitopes have never been characterized, it is tempting to speculate that alternative MPO epitopes, other than MPA ones, develop in ANCA-positive EGPA, thus contributing to a mitigated vasculitic phenotype.

Therefore, stronger efforts should be made to better characterize the mechanisms underlying EGPA pathogenesis. Further studies are required in order to molecularly characterize clinical phenotypes by taking into account complex -omics data (e.g., genomics, epigenomics, transcriptomics, proteomics, and metabolomics). Shifting the subject of EGPA research from clinical phenotype to molecular endotype would possibly allow to identify valuable biomarkers and therapeutic targets that could improve diagnostic precision and therapeutic outcomes.

## Author Contributions

FF and FB equally contributed to the conception and design of the work and drafted the article. GE critically revised the article. All authors approved the final version of the manuscript.

## Conflict of Interest

The authors declare that the research was conducted in the absence of any commercial or financial relationships that could be construed as a potential conflict of interest.
